# Multiple regimes of operation in bimodal AFM: understanding the energy of cantilever eigenmodes

**DOI:** 10.3762/bjnano.4.45

**Published:** 2013-06-21

**Authors:** Daniel Kiracofe, Arvind Raman, Dalia Yablon

**Affiliations:** 1 Purdue University School of Mechanical Engineering, West Lafayette, IN, USA; 2 ExxonMobil Research and Engineering Co., Annandale, NJ, USA

**Keywords:** atomic force microscopy, bimodal AFM, cantilever eigenmodes, polymer characterization

## Abstract

One of the key goals in atomic force microscopy (AFM) imaging is to enhance material property contrast with high resolution. Bimodal AFM, where two eigenmodes are simultaneously excited, confers significant advantages over conventional single-frequency tapping mode AFM due to its ability to provide contrast between regions with different material properties under gentle imaging conditions. Bimodal AFM traditionally uses the first two eigenmodes of the AFM cantilever. In this work, the authors explore the use of higher eigenmodes in bimodal AFM (e.g., exciting the first and fourth eigenmodes). It is found that such operation leads to interesting contrast reversals compared to traditional bimodal AFM. A series of experiments and numerical simulations shows that the primary cause of the contrast reversals is not the choice of eigenmode itself (e.g., second versus fourth), but rather the relative kinetic energy between the higher eigenmode and the first eigenmode. This leads to the identification of three distinct imaging regimes in bimodal AFM. This result, which is applicable even to traditional bimodal AFM, should allow researchers to choose cantilever and operating parameters in a more rational manner in order to optimize resolution and contrast during nanoscale imaging of materials.

## Introduction

Atomic force microscopy (AFM) has arisen as one of the key tools for characterization of morphology and surface properties of materials (e.g., polymer blends and composites) at the micro-/nanoscale [1]. Although there are many different operating modes in AFM, one of the most popular is amplitude modulation (AM-AFM), commonly known as tapping mode, in which the cantilever is oscillated at its first natural frequency. AM-AFM provides two basic images of the surface, a height (topography) image and the so-called “phase” image. The latter is related to material properties and is frequently used to distinguish different domains or different blend components from one another. While phase imaging often provides good contrast between different materials, it is difficult to determine the exact mechanical property that is responsible for a particular contrast. Further, the contrast is sometimes poor between distinct domains or components within a polymer blend or composite. Finally, artifacts induced by bistable imaging in attractive and repulsive regimes often confuse the interpretation of phase images.

An extension of AM-AFM called bimodal AFM [2], a capability that has been applied to a variety of materials over the past five years, can overcome some of these limitations. Bimodal AFM oscillates the AFM cantilever at two frequencies simultaneously. This adds two additional channels of information beyond the standard AM-AFM method, namely, the amplitude and phase at the second frequency, which can be used to enhance contrast between materials. Further, this information can be assigned to specific types of interactions (i.e., conservative/elastic versus dissipative) [3]. With its recent widespread usage, especially among soft materials such as biological materials and polymers, bimodal AFM has demonstrated its capability to provide new contrast and information in the higher order mode [4–8].

The traditional choice in bimodal AFM is to oscillate the cantilever at its first two natural frequencies, namely the first and second flexural eigenmodes of the cantilever. However, modern AFMs have the frequency bandwidth to excite the third, fourth, or even fifth flexural eigenmode. This allows for the fundamental eigenmode to be paired with many different higher order eigenmodes for bimodal AFM operation (or even multiple higher eigenmodes simultaneously [9]). In this work, we wish to examine the choice of specific higher order eigenmodes for bimodal operation in order to understand if they provide any practical advantages in terms of material discrimination and identification. There are several questions of interest. First, do higher order eigenmodes probe the same types of tip–sample interactions as lower order eigenmodes? For example, polymers may be viscoelastic, so there could be a different interaction due to the frequency difference. Second, do higher order eigenmodes provide better contrast between materials and/or higher image quality? Third, can any rational guidance be provided for the selection of the higher order mode as well as operating parameters (e.g., drive amplitudes, setpoints, etc.) in order to obtain the most meaningful interpretation from the images?

In this work we show a series of bimodal experiments on a multicomponent polymer blend using different combinations of eigenmodes. Bimodal AFM shows excellent contrast between the different components. We will show that there are several interesting effects that depend on the choice of eigenmodes and operating parameters, which suggest that there are actually at least three distinct operating regimes in bimodal AFM (akin to attractive/repulsive regimes in AM-AFM). Numerical simulations are then used to provide further insight into these different regimes.

## Experiment

### Methods

An AFM (Asylum Research, Santa Barbara, CA) with a high frequency cantilever holder is used. Experiments were conducted using silicon Olympus cantilevers. Three different models were tried – AC240, AC200, and AC160 – which have nominal stiffness values of approximately 2, 9, and 26 N/m. Similar results were obtained for all cantilever models, and representative results for AC200 cantilevers are shown here.

The optical lever sensitivity (also known as “invOLS”) of the first, second, and third eigenmodes was obtained from dynamic approach curves on a mica surface (in repulsive regime on a stiff surface, the amplitude decreases by approximately 1 nm when the *z*-piezo is displaced by 1 nm [10]). The sensitivity of the fourth eigenmode could not be obtained in this way because the modal stiffness was too high (relative to the tip–sample contact stiffness). Therefore the fourth eigenmode sensitivity was estimated based on Euler–Bernoulli beam theory and the data for the lower order modes. The stiffness of each eigenmode was calibrated by using the thermal tune method [11–12]. The thermal response of the fourth eigenmode was sometimes too small to give a meaningful calibration, and in this case the stiffness was estimated from beam theory. The natural frequency ω_4_, however, can be measured precisely, and the ratio ω_4_/ω_3_ is within 7% of the value predicted by beam theory, suggesting that the stiffness should not be too far from beam theory predictions either.

Care was taken to tune the driving frequency exactly to the natural frequency before every experiment. The effects of squeeze film damping [13] are such that the phase can change by an appreciable amount (10 degrees) when the cantilever is moved a few micrometers away from the surface. Further, piezo resonances can distort the tuning curve. For plain AM-AFM at the first natural frequency, piezo resonances are generally only an issue in liquid [14]. However, on our instrument, piezo resonances can distort the higher eigenmode tuning curves significantly, especially for third and higher eigenmodes. Therefore, a thermally driven spectrum was obtained when the cantilever was positioned approximately 100 nm above the surface. A curve fit to the thermal spectum was used to determine the natural frequency [14]. The drive frequency was then set to this frequency, and the phase (lag) offset was set to 90 degrees.

The sample used was a ternary polymer blend consisting of isotactic polypropylene (PP, ExxonMobil Chemical Company), high density polyethylene (PE, ExxonMobil Chemical Company), and polystyrene (PS, Polysciences). A blend of 3:1:1 (by mass) of PP/PE/PS was prepared in a Brabender mixer (Brabender Instruments, South Hackensack, NJ) at 180 °C, 60 rpm, and 5 min of mixing and then compression molded into a bar. This was then cryo-cut using a microtome (Ultracut 6, Leica Mikrosysteme GmbH, Vienna, Austria) at −120 °C with a glass and a diamond knife prior to AFM imaging. This sample was chosen because the individual components are well characterized and can be easily distinguished in AFM images based on morphology, surface roughness, and height. Specifically, the matrix (dominant component) is polypropylene, with approximately circular polyethylene and polystyrene domains. The polyethylene domains appear rough because of the lamellar structure, and the polystyrene domains show fracture marks from the cryomicrotoming. Dynamic mechanical analysis using time-temperature superposition was performed on each component individually by using the method described in [15].

### Results

**Figure 1:**
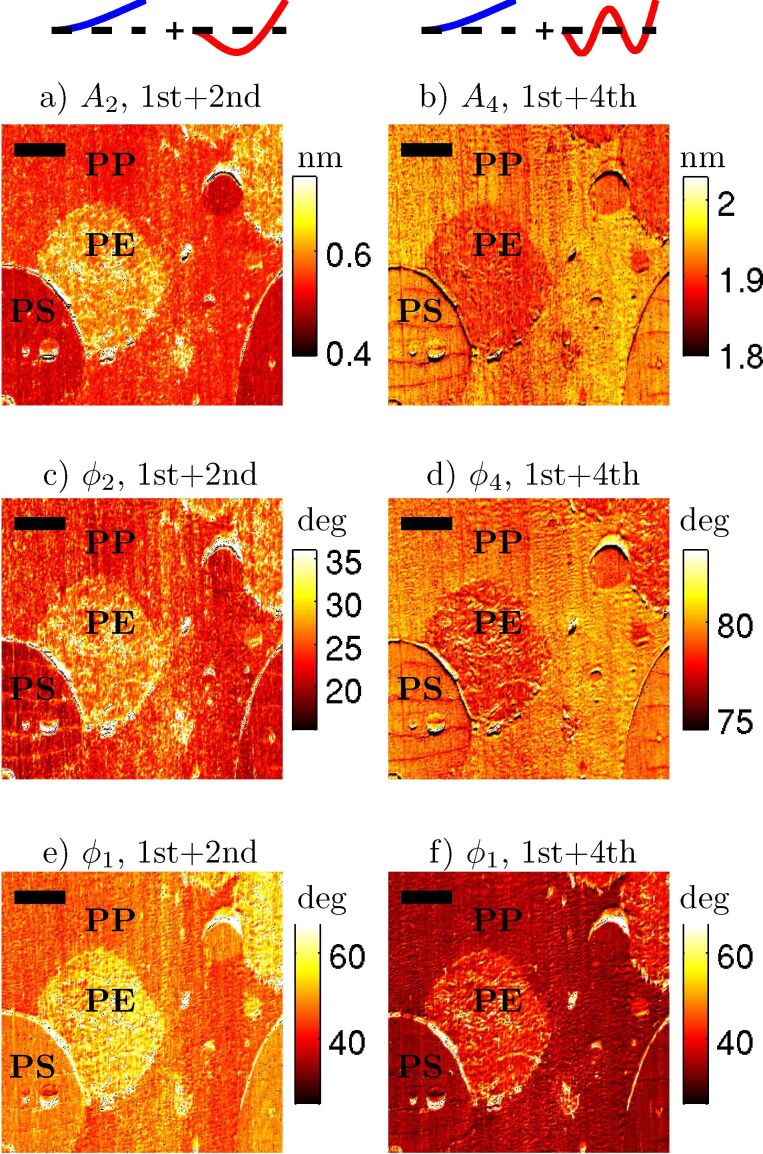
An 11 × 11 micrometer scan of a three component polymer blend (PS, PP, PE) imaged with an Olympus AC200 cantilever in two different modes: The left column (a,c,e) is “classical” bimodal AFM with the 1st and 2nd natural frequencies excited, and the right column (b,d,f) is bimodal AFM with the 1st and 4th natural frequencies excited. The eigenmode shapes are drawn above each column. The free amplitudes were the same in both cases (50 nm at the 1st eigenmode, and 2.5 nm at the 2nd or 4th eigenmode, respectively). Comparing the polyethylene (PE) to the other components, both the amplitude (a,b) and the phase lag (c,d) have a contrast between the modes. For the “1st + 2nd” image, PE has the highest *A*_2_ and 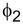, but for the “1st + 4th” image, PE has the lowest *A*_4_ and 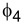. 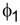 (e)(f) also shows a distinct different between the two imaging modes. The scale bar is 2 μm.

The first experiment that was carried out was to compare two scans where all parameters were the same except for the choice of higher eigenmode (e.g., “1st + 2nd” eigenmodes versus “1st + 4th”). The objective was to determine if the two scans showed the same type of contrast between the components, and to determine if one scan showed better contrast or higher resolution. Typical results of bimodal imaging with various higher order modes on the ternary blend sample are shown in Figure 1. The cantilever parameters were *k*_1_ = 4 N/m, *Q*_1_ = 212 (remaining parameters given in Table 1). Two different scans are compared. The first scan is a bimodal image using the 1st and 2nd eigenmodes (left column (a,c,e)), and the second scan is a bimodal image using the 1st and 4th eigenmodes (right column (b,d,f)). In both cases, the free amplitude of the 1st eigenmode was 50 nm, the setpoint was 50%, and the free amplitude of the higher eigenmode (either 2nd or 4th) was 2.5 nm. There are several interesting differences between the “1st + 2nd” bimodal image and the “1st + 4th” bimodal image. The first observation involves relative contrast between the three different materials present in the blend. The contrast between the PP and PS domains are very similar in all the images. However, the contrast between the PE domain and the PP or PS domains changes. When considering the “1st + 2nd” scan (left column), the polyethylene has a higher (brighter) amplitude (a) and phase lag (c) than either the polystyrene or the polyproylene. However, in the “1st + 4th” scan (right column) the contrast is exactly reversed. The polyethylene has a lower (darker) amplitude (b) and phase (d) than either the polystyrene or the polypropylene.

**Table 1:** Calibrated cantilever parameters for the experiments.

mode	1	2	3	4

stiffness (N/m)	4	78.5	366	1330^a^
quality factor	212	457	507	600^a^
natural frequency (kHz)	117	674	1758	3235

^a^Values are estimates.

A second observed difference between the bimodal images in “1st + 2nd” eigenmode versus “1st + 4th” eigenmode occurs in the first eigenmode phase (lag) channel. The overall first eigenmode phase (lag) has decreased considerably (darker) between the “1st + 2nd” scan (e) and the “1st + 4th” scan (f). The change in the first eigenmode phase is surprising because the bimodal AFM literature has generally treated the second frequency as an independent channel that provides additional information but does not affect the response of the first eigenmode [2,5,16–17]. The argument is that the first eigenmode is unaffected by the higher frequency oscillation because the first eigenmode amplitude is more than an order of magnitude larger than the higher eigenmode amplitude. For example, in [8] it was recently demonstrated experimentally that when *A*_1,free_/*A*_2,free_ = 10 : 1 (or greater) there is no apparent coupling between the 1st and 2nd eigenmodes, but that when *A*_1,free_/*A*_2,free_ = 1 : 1 there is a coupling between the eigenmodes. However, in Figure 1f, we are using a ratio of *A*_1,free_/*A*_4,free_ = 20 : 1, but yet we see a strong change in the first eigenmode, indicating some coupling between the eigenmodes. Therefore, we conclude that a large amplitude ratio is a necessary but not sufficient condition for the two eigenmodes to be uncoupled. Later, we will attempt to determine a sufficient condition for the eigenmodes to be uncoupled.

These two features, a contrast reversal between PE and PP in the higher eigenmodes and an overall drop in the first eigenmode phase, were repeatable across multiple different cantilevers on different days, on different cantilever models with stiffness from 2 to 26 N/m, on different locations on the sample, and on different samples. Similar results to the “1st + 4th” higher order eigenmode amplitude contrast reversal and lowering of first order eigenmode phase were also observed in “1st + 3rd” bimodal imaging. We chose to focus on “1st + 4th” imaging instead of “1st + 3rd” because the 4th eigenmode was more novel experimentally.

We first discuss the PE/PP contrast reversal in the higher eigenmodes. Broadly speaking, we could imagine two possible explanations for these results. First, it could be that the tip–sample interaction probed by the fourth eigenmode is significantly different to the interaction probed by the second eigenmode (e.g., due to viscoelasticity). Alternatively, it could be that there is a difference in the cantilever dynamics at the fourth eigenmode such that it responds to the exact same tip–sample interaction in a different way. Next we show experiments designed to distinguish between these two possibilities.

Regarding the second possibility, it was pointed out in [18] that in bimodal AFM, the fundamental quantity may not be the ratio of the amplitudes of the two eigenmodes, but rather the ratio of the energy of the two eigenmodes. In our case, we compared the “1st + 2nd” scan and the “1st + 4th” scan at the exact same free amplitudes. But because *k*_4_ > *k*_2_, they were not compared at the same energy levels. Understanding the energy flow while tapping on the sample can be complicated (because of energy transfers between eigenmodes analogous to [19]). Therefore, as a first approximation, consider the kinetic energy of a freely vibrating (no tip–sample interaction) cantilever eigenmode, which is 
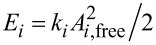
 (both kinetic and potential energy give the same result; derivation in Supporting Information File 1). For the conditions under which the data in Figure 1 were collected, *E*_1_/*E*_2_ = (4(50^2^))/(78.5(2.5^2^)) = 20.4. So the first eigenmode has much more energy than the second eigenmode. But when comparing the first and fourth eigenmodes we find *E*_1_/*E*_4_ = (4(50^2^))/(1330(2.5^2^)) = 1.2. Therefore the fourth eigenmode actually has approximately the same energy as the first eigenmode.

**Figure 2:**
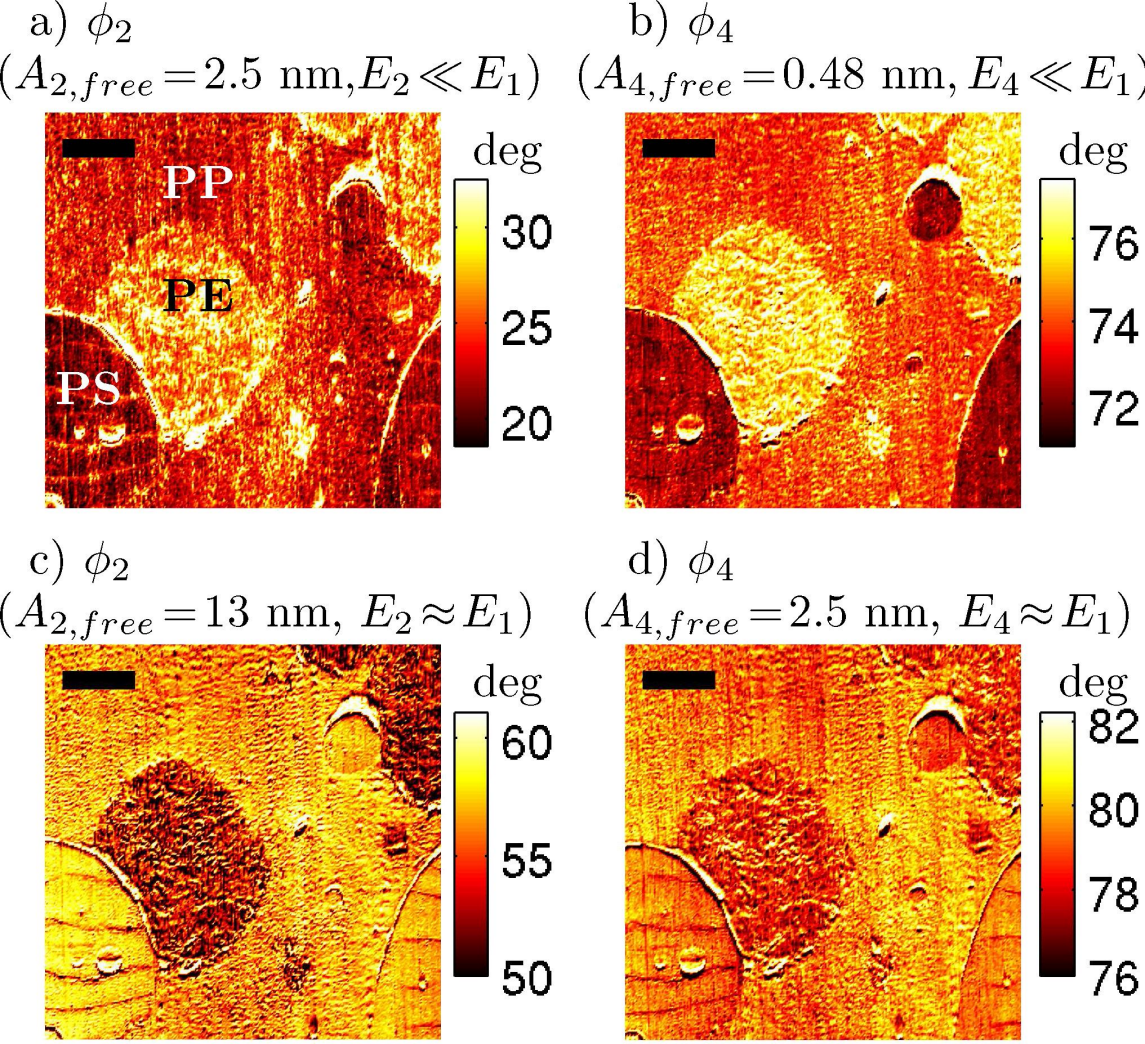
Using the same cantilever and sample from Figure 1, the imaging modes are compared at different energy levels. In the top row (a, b) the first eigenmode has a larger energy than the higher mode (2nd or 4th respectively). In the bottom row (c, d), the first eigenmode energy is comparable to the higher eigenmode energy. In the top row, the PE has the highest phase lag, whereas in the bottom row the PE has the lowest phase lag. This shows that the contrast reversal observed in Figure 1 is not caused by the choice of eigenmode alone, but by the energy in the eigenmode. The scale bar is 2 μm.

To evaluate which effect the eigenmode energy has on the results, we repeat the experiment except this time we consider not just the choice of eigenmode, but also adjust the drive amplitudes to control the eigenmode energy level. This leads to four bimodal scans: Figure 2a “1st + 2nd” modes with *E*_2_ << *E*_1_; Figure 2b “1st + 4th” modes with *E*_4_ << *E*_1_; Figure 2c “1st + 2nd” with *E*_2_ ≈ *E*_1_; and Figure 2d “1st + 4th” modes with *E*_4_ ≈ *E*_1_. The scans in (a) and (d) were taken under the same conditions as those in Figure 1. It is clear that the PE/PP contrast is similar between the “1st + 2nd” and “1st + 4th” cases as long as the energies are kept the same. That is, for *E*_2_ << *E*_1_ or *E*_4_ << *E*_1_, PE is bright with respect to PP as shown in Figure 2a and Figure 2b. For *E*_2_ ≈ *E*_1_ or *E*_4_ ≈ *E*_1_, PE is dark with respect to PP as shown in Figure 2c and Figure 2d. This suggests that the contrast reversal is due solely to the relative eigenmode energy levels, and not to the choice of eigenmode.

**Figure 3:**
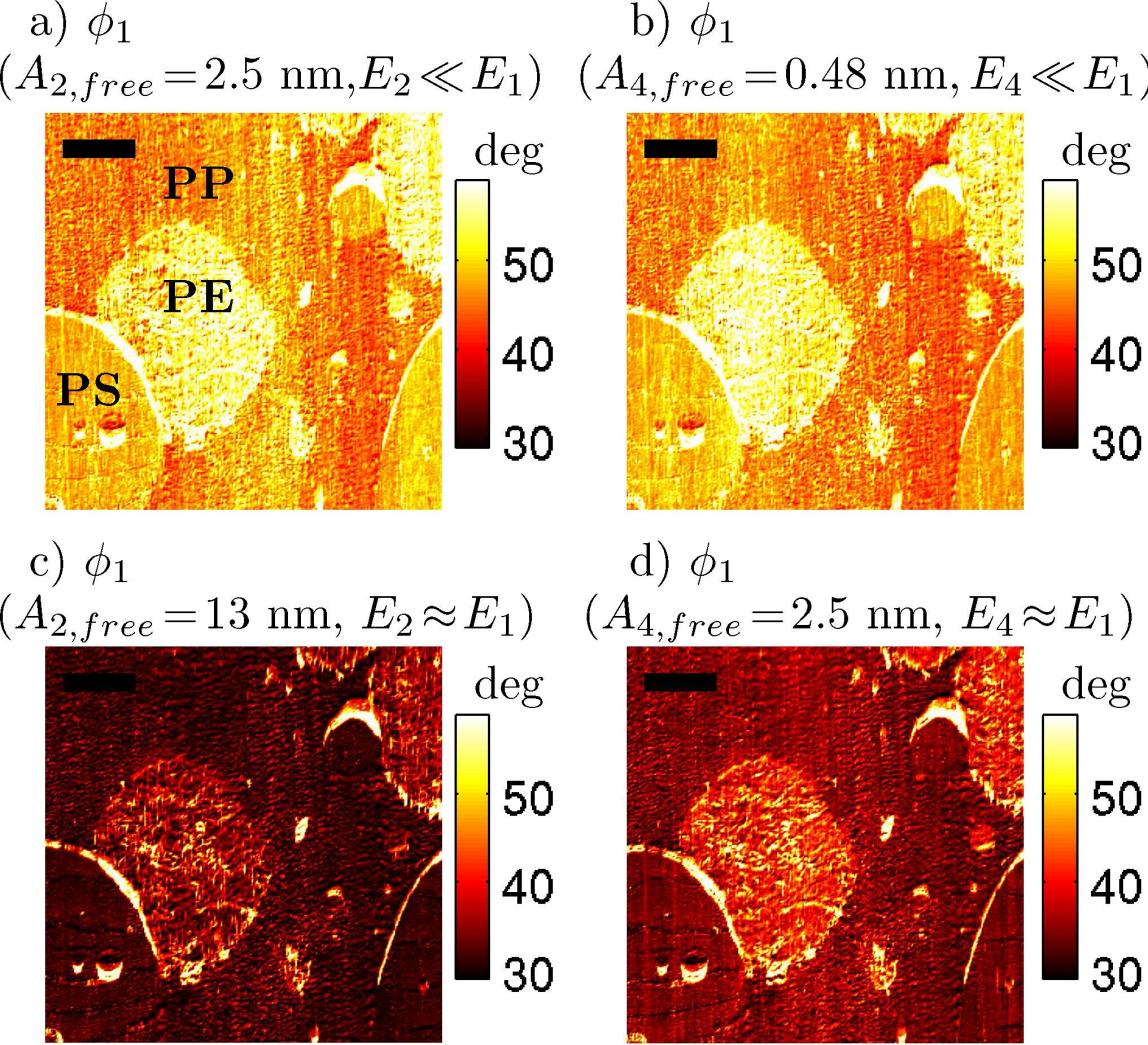
The same experiment as Figure 2, plotting first eigenmode phase lag 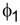. In the top row (a, b) the first eigenmode has a larger energy than the higher mode (2nd or 4th, respectively). In the bottom row (c, d), the first eigenmode energy is comparable to the higher eigenmode energy. The first eigenmode phase drops considerably between the top and bottom rows, indicating that in the high energy state there is a nonlinear coupling between the eigenmodes that is affecting the first eigenmode. This is clearly due to the energy ratios, and not the choice of higher eigenmode (i.e., little difference between left and right columns). The scale bar is 2 μm.

Further, in Figure 3, it can be seen that the first eigenmode phase (lag) shows the same pattern: the drop in phase is due to the relative eigenmode energy levels. When the relative energy in the first eigenmode dominates (a and b), 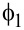 is relatively high, but when the two eigenmodes have about the same energy (c and d), then 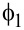 is relatively low. How can this change in phase be interpreted? The meaning of phase angles in AM-AFM is a difficult topic that is frequently misinterpreted. In AM-AFM imaging, it is not possible to separately identify the conservative (e.g., elastic) and dissipative (e.g., viscous) components of the tip–sample interaction by using the first eigenmode phase (in contrast to FM-AFM imaging, or higher eigenmode imaging in bimodal AFM). Instead, the first eigenmode phase gives information about the ratio of the dissipative interaction to the conservative interaction. The lower phase lag in (c) and (d) indicates a lower ratio of dissipative interaction to conservative interaction (i.e., either less dissipation, higher conservative forces, or both), as compared to (a) and (b). It appears that there are two distinct operating regimes in bimodal AFM, which have distinctly different responses to material property contrast and distinctly different energy dissipations in the first eigenmode. In the next section, numerical simulation is used to provide further insight into this second regime.

## Simulation

### Modeling

In order to provide insight into the physical processes at work, we use numerical simulations. The VEDA simulator (a freely available, open-source, web-based [20] AFM simulator developed by the authors) is used for numerical simulation. A full description of the simulator is given in [21–22]. Here we review the features relevant to the present work. The modeling starts with the Euler–Bernoulli partial differential equation for deflections of a slender, rectangular cantilever beam in a ground-fixed inertial frame, subject to a hydrodynamic damping force, a driving force, and a tip–sample interaction force

[1]
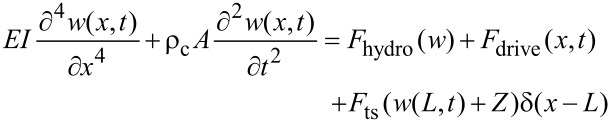


where *E*, *I*, ρ_c_, *A*, *w*, *x*, *t*, *F*_hydro_, *F*_ts_, *F*_drive_ and δ are the cantilever Young’s modulus, area moment of inertia, density, cross-sectional area, deflection, axial coordinate, time, hydrodynamic force, tip–sample interaction force, driving (excitation force), and Dirac delta, respectively. The hydrodynamic forces are converted into an effective modal viscosity and added mass [23], and then the equation is discretized in the basis of cantilever eigenmodes by Galerkin’s method following [24]. The method is to write *w* as


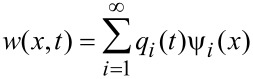


where ψ*_i_*(*x*) is the *i*th eigenmode shape and *q_i_*(*t*) is referred to as a modal coordinate. ψ is chosen such that ψ*_i_*(*L*) = 1 so that the modal coordinates are the deflection of the cantilever at the free end. This scaling is important because it allows the calibrated stiffnesses of the eigenmodes to be incorporated directly into the model [24]. An approximation is made by keeping only the first *N* eigenmodes. We take *N* = 4 in this work. This reduces the original equation to a set of four ordinary differential equations:

[2]
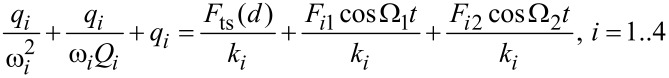


where *q_i_*(*t*), ω*_i_*, *Q_i_*, and *k_i_*, are the tip deflection, natural frequency (rad/s), quality factor, and equivalent stiffness, respectively, and


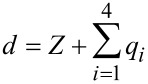


is the tip–sample gap, where *Z* is the cantilever–sample separation. A bimodal excitation is used where Ω_1_ is the first driving frequency, Ω_2_ is the second driving frequency, *F_i_*_1_ is the force on the *i*th eigenmode due to the first excitation and *F_i_*_2_ is the force on the *i*th eigenmode due to the second excitation. In this work, we take Ω_1_ = ω_1_ and Ω_2_ = ω_2_ to simulate bimodal driving of the 1st and 2nd eigenmodes.

The tip–sample interaction force *F*_ts_(*d*) is described by a modified DMT model that includes a term for surface energy hysteresis. In other words, the force when the tip is approaching the sample is different from the force when the tip is retracting from the sample. The model is based on the one proposed by [25] and is described in detail in Supporting Information File 1.

### Simulations Results

**Figure 4:**
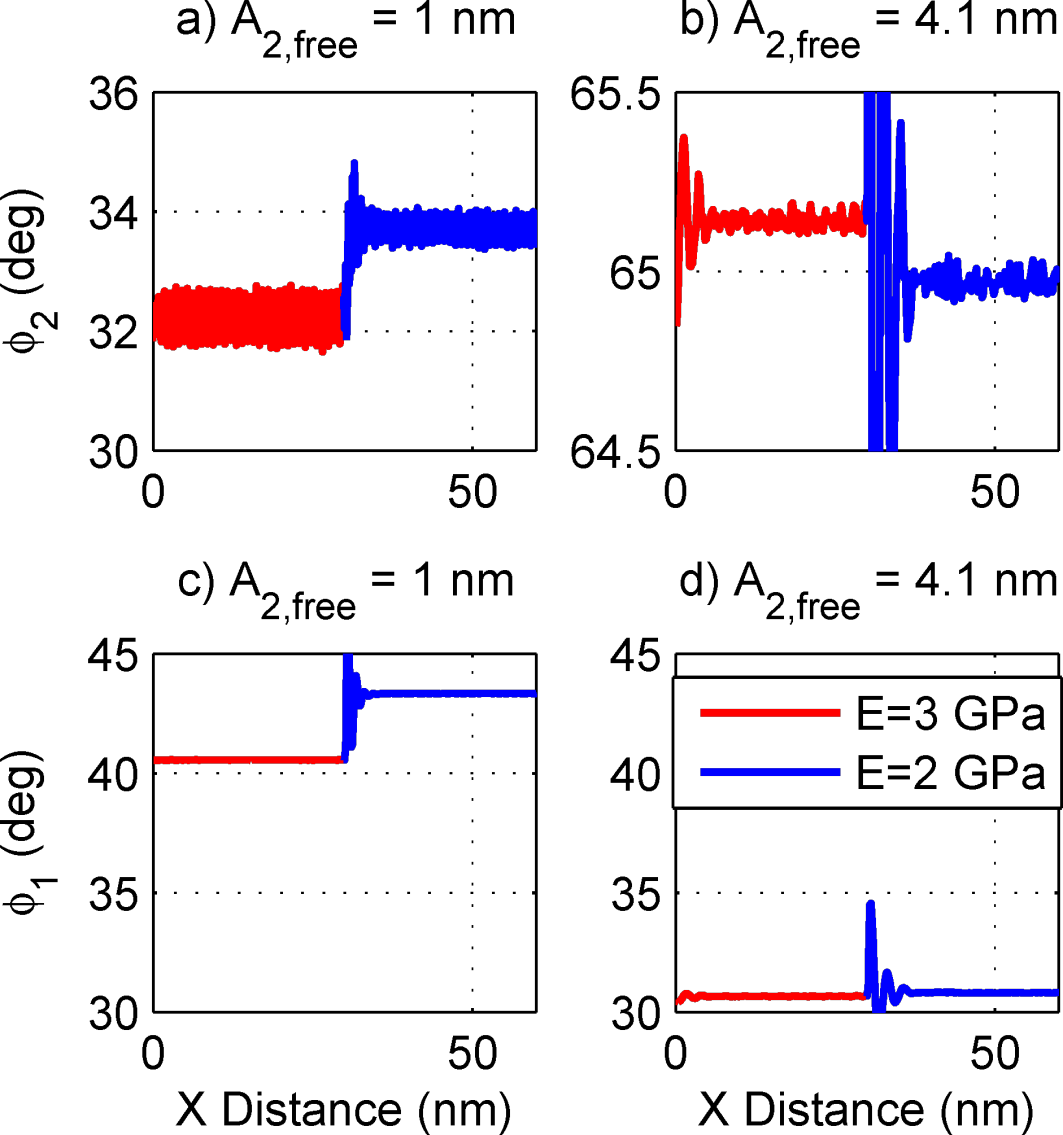
Simulated line scans for the parameters in Table 2 and two different second eigenmode drive amplitudes. The simulated sample is a PE domain in the center of a PP matrix. Comparing (a) versus (b), there is a clear contrast reversal of 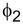 between the low and high second eigenmode drive. This matches the experimental observations of Figure 2. Further, comparing (c) versus (d), 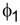 drops as *A*_2,init_ is raised. This matches the experimental observation in Figure 3.

Figure 4 shows two simulations of a line scan in AM-AFM. The line scan crosses over two different materials that are located side by side. The material on the left (red lines) has a Young’s modulus of 3 GPa and the one on the right (blue lines) has a modulus of 2 GPa. These values are close to the storage modulus from dynamic mechanical analysis (time–temperature superposition was used to obtain the value at 250 kHz) for polypropylene and polyethylene at 250 kHz, respectively. Both materials have a surface energy hysteresis term of 0.06 J/m^2^ (chosen to approximately match the average energy dissipation in AM-AFM experiments). The same first eigenmode amplitude is used for both simulations, while two different drive amplitudes are chosen for the second eigenmode: (b) and (d) show a larger amplitude for which *E*_2_ ≈ *E*_1_; (a) and (c) show a smaller amplitude for which *E*_2_ << *E*_1_. These conditions were chosen to approximately match the experiments. The full parameters for the simulation are given in Table 2.

**Table 2:** Simulation parameters. Hamaker constant and surface energy are tuned to match the experiment. All other values are measured or nominal values.

	mode 1	mode 2

stiffness (N/m)	4	160
quality factor	200	400
natural frequency (kHz)	10	344
driving frequency (kHz)	10	344
free amplitude (nm)	40	varies
setpoint ratio	50%	
sample modulus (GPa)	2–3	
van der Waals adhesion force (nN)	1.4	
tip radius (nm)	10	
intermolecular distance (nm)	0.2	
surface energy change (J/m^2^)	0.06	

The results in Figure 4 qualitatively match the features in the experiment. Specifically, for the smaller amplitude (1 nm, a and c), the second eigenmode phase (lag) on the softer (blue) material is higher. But for the larger amplitude (4.1 nm, b and d), the contrast between the two materials reverses and the second eigenmode phase (lag) on the softer (blue) material is lower. This contrast reversal qualitatively matches the experimentally observed contrast reversal from Figure 2. Also, the first eigenmode phase drops significantly for the larger second eigenmode amplitude, as shown in Figure 4c and Figure 4d, which is exactly the trend noted in the experiments in Figure 3.

**Figure 5:**
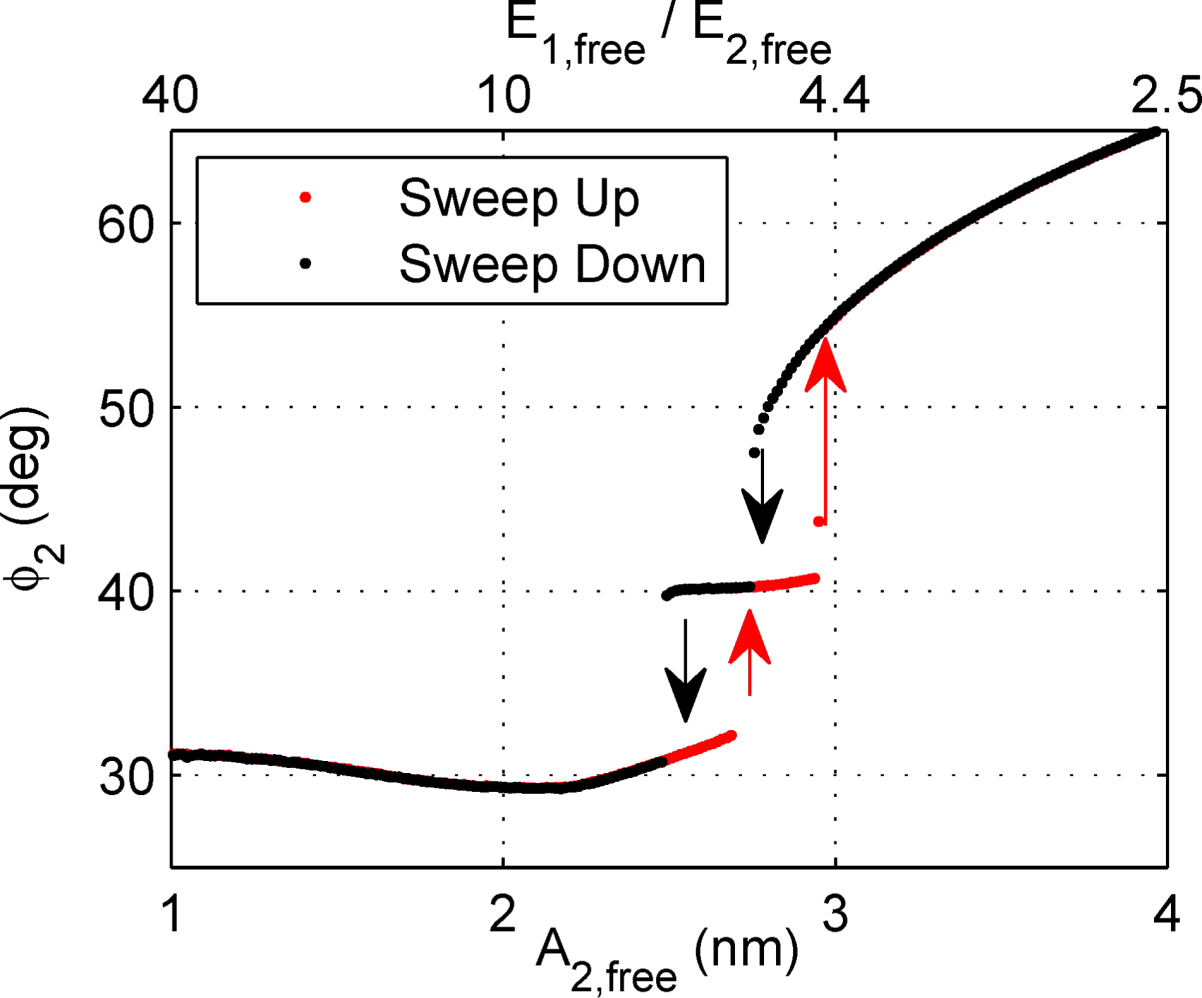
A simulation in which the second eigenmode drive amplitude is swept up and then down continuously. The feedback controller remains active so that a constant setpoint is maintained (i.e., *A*_1_ is constant). The parameters are given in Table 2. The lower *x*-axis shows the second eigenmode amplitude and the upper *x*-axis the ratio of the first and second eigenmode energies (both quantities calculated based on the free case). Interestingly, there are two discrete jumps in the second eigenmode response indicating that there are three distinct dynamic states. Further, the jumps do not occur at the same amplitude on sweep up versus sweep down, indicating that the states are bistable.

To further explore this phenomenon, we perform a simulation in which the cantilever is tapping on a surface with the normal feedback controller on while the second eigenmode drive amplitude (and hence second eigenmode energy) is slowly increased from zero to a maximum amplitude and then slowly decreased back to zero, as shown in Figure 5. There are two abrupt jumps in the response as the drive is changed. This plot suggests that there are not two but three distinct operating regimes in bimodal AFM, depending on the amplitudes of the eigenmodes. Further, there is a hysteresis, i.e., the jump up and jump down do not happen at the same amplitude, indicating the presence of a bistability. Two different states are possible for the same combination of parameters (similar to the attractive/repulsive bistability in conventional AM-AFM).

**Figure 6:**
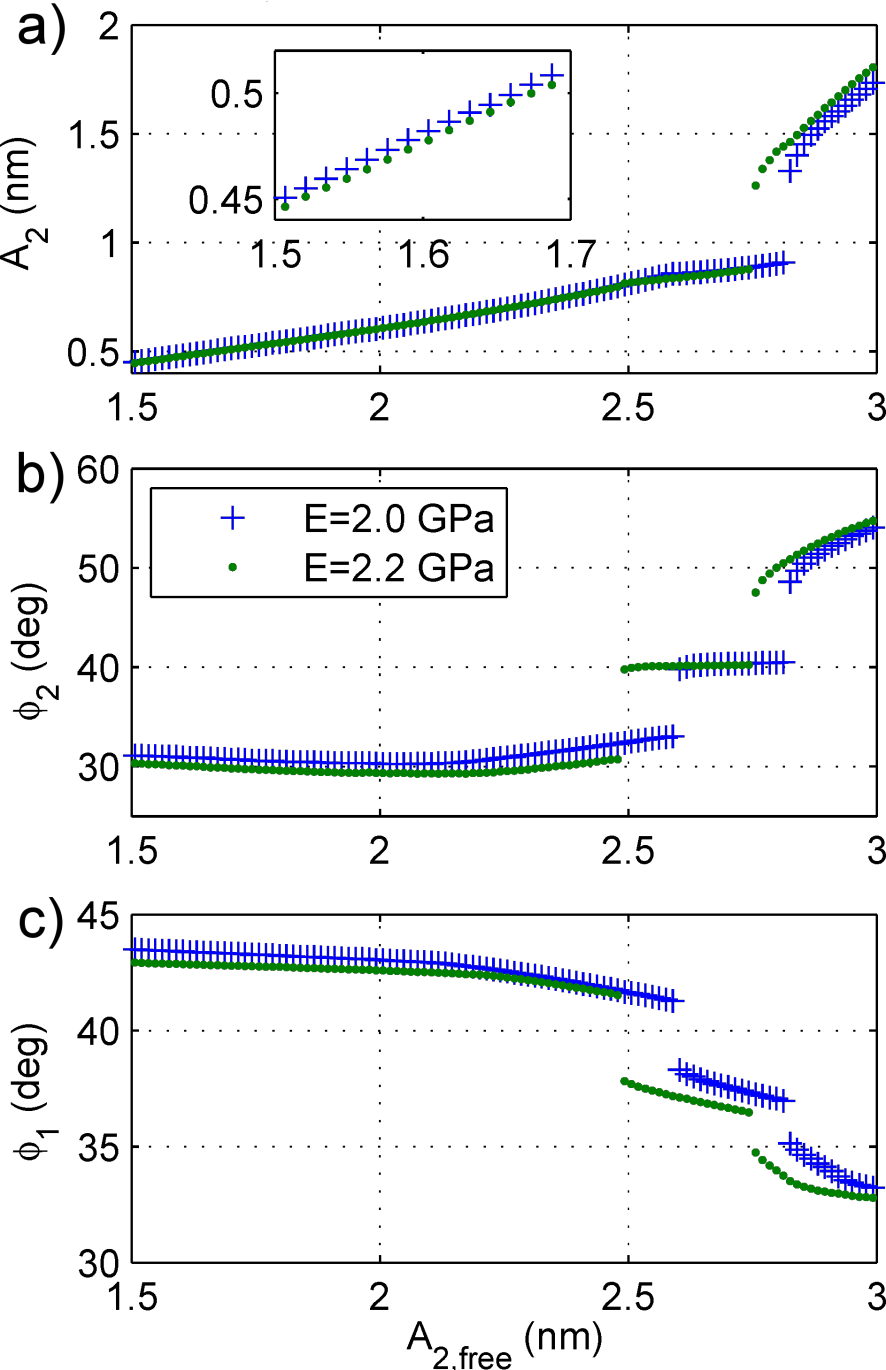
A drive amplitude sweep similar to Figure 5 except that two different materials are compared (both are sweeps from high amplitude down to low amplitude). The transitions between the different states happen at slightly different amplitudes for each material. It is clear from (a) and (b) that the contrast reversal observed in Figure 1, Figure 2, and Figure 4 is caused by the transition from one state to another. That is, in the state on the left (low *A*_2,init_), *A*_2_ and 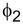 on the stiffer material are higher than *A*_2_ and 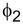 on the softer material. But, in the state on the right (high *A*_2,init_), *A*_2_ and 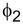 on the stiffer material are lower than *A*_2_ and 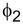 on the softer material. The middle state appears to have very little contrast between the two materials. Finally, 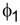 drops as *A*_2,init_ is raised, with big drops at each state transition.

A question that naturally arises is whether the amplitude and phase contrast reversals observed earlier have any correlation with the jumps between the different states. To this end, in Figure 6 amplitude sweeps are shown on two different materials (in this case, *E* = 2.0 GPa and *E* = 2.2 GPa, and the drive is swept from a high amplitude down to a low amplitude, corresponding to the range of amplitudes used in Figure 4). In fact, the contrast reversal correlates exactly to the different states. In the left state (lower *A*_2,free_), both *A*_2_ (Figure 6a) and 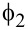 (Figure 6a) on the stiffer material are *lower* than on the softer material. In the right state (higher *A*_2,free_), both *A*_2_ and 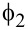 on the stiffer material are *higher* than on the softer material. In the middle state, there is not much differentiation between the materials. Note that the jumps between states happen at a slightly different drive amplitude depending on the sample modulus. This is the reason that *E* = 2.0 GPa and *E* = 2.2 GPa were picked for this simulation. For *E* = 2.0 GPa versus *E* = 3.0 GPa, the jumps happen at very different amplitudes and it is more difficult to make a comparison. Because there is less contrast in modulus (as compared to the experiment), there is less contrast in phase in Figure 6 than observed in the experiment. Furthermore, Figure 6c shows a drop of 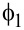 as the eigenmode amplitude (and hence energy) is increased as well. This is consistent with the experimental observations in Figure 3.

**Figure 7:**
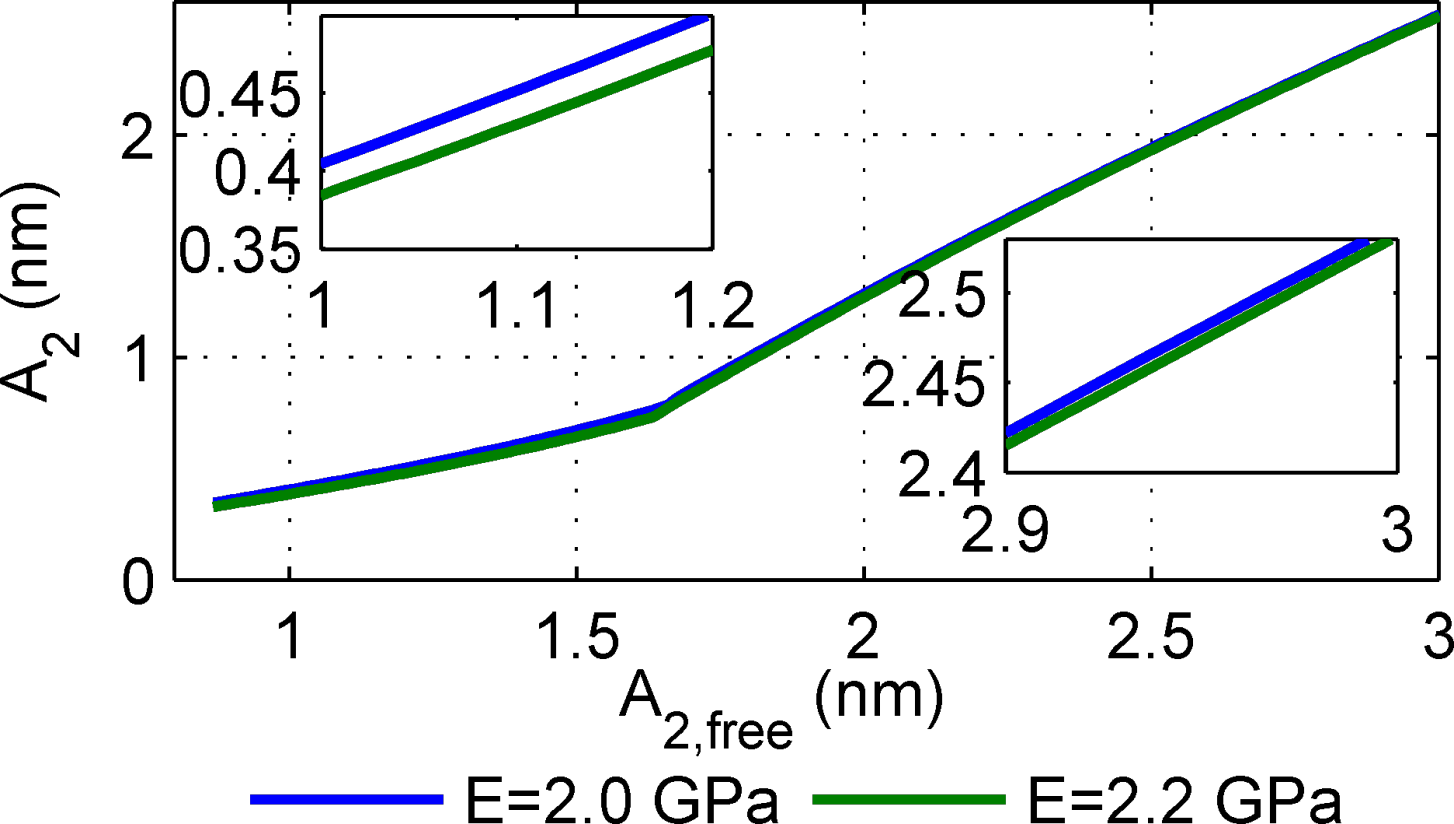
The simulation of Figure 6 repeated except with a pure Hertz model (no energy dissipation). In comparison to Figure 6, there are no sudden jumps and the second eigenmode contrast does not reverse.

The fact that the cantilever dynamics behave differently depending on the ratios of first and second eigenmode energy has been previously suggested [18]. However, that work considered only a homogenous sample, so the possibility of contrast reversal was not considered. Further, the fact that there are three states separated by a discontinuous jump was not considered. Why the contrast should reverse between the states is not immediately obvious, but it clearly happens in both experiment and simulation. To provide further insight, the simulation of Figure 6 is repeated but with a purely conservative tip–sample interaction (i.e., Hertz contact without energy dissipation). The result is shown in Figure 7. In this case there are no discontinuous jumps. There is a point at which the slope of the amplitude and phase curves change, but there is no contrast reversal in either. Also, there is essentially no change in the first eigenmode phase (not shown).

## Discussion

From a practical point of view, it is not immediately obvious if bimodal imaging using the higher states is advantageous or not. On the one hand, for certain combinations of parameters/materials, the material contrast (i.e., percent change in amplitude for a given change in Young’s modulus) can be an order of magnitude higher in these states than in standard bimodal imaging. For example, in Figure 6a, the two materials are essentially indistinguishable for *A*_2,free_ < 2.5 nm, but are very clearly separated for *A*_2,free_ > 2.8 nm. This is consistent with a previous report [8] that suggested better contrast on polymers might be achieved with higher *A*_2,free_. On the other hand, there is bistability between the different regimes. This may cause difficulties in imaging, as the attractive/repulsive regimes in conventional AM-AFM do. It may be possible to overcome the bistability by using frequency modulation, phase modulation or other newer feedback control schemes, such as drive modulation.

From a theoretical point of view, more research is needed to understand the nature of the different states and exactly why the contrast should reverse. The fact that there is no contrast reversal for the elastic case in Figure 7 suggests that the tip–sample energy dissipation plays a key role in the contrast reversal. Presumably, an energy transfer between the eigenmodes is involved.

Practically, this result reinforces the suggestion of Stark [18] that energy ratios and not amplitude ratios are the important quantity to consider in bimodal AFM. Researchers using AFM will be able to select operating conditions more intelligently if they calculate the energy ratios involved instead of amplitude ratios.

Finally, the importance of energy ratios highlights the need for better methods to calibrate stiffness and optical lever sensitivity of higher order eigenmodes. The current state of the art works well for the first few eigenmodes but becomes less reliable for third and higher modes. As multifrequency AFM evolves toward quantitative measurements using higher order eigenmodes, interferometer based AFMs, which do not suffer from these calibration problems, may become more attractive than optical lever (photodiode) based AFMs.

## Conclusion

We have shown experimentally that there are multiple distinct imaging regimes in bimodal AFM. The different states were identified by contrast reversals on a multicomponent polymer blend. Higher eigenmode bimodal AFM (e.g., “1st eigenmode + 4th eigenmode”) behaves essentially the same as traditional bimodal AFM (“1st + 2nd”), when operated at similar energy levels. When the energy of the higher eigenmode is much smaller than the energy of the first eigenmode, then the two eigenmodes are essentially uncoupled. This is the regime that the majority of classical bimodal AFM studies have explored. When the energy of the higher eigenmode is comparable to the energy of the first eigenmode, there are additional distinct imaging regimes involving coupling between the eigenmodes. We have shown that the experimentally observed contrast reversals can be qualitatively predicted by the use of numerical simulation. Further, the numerical simulation has shown that there are actually three distinct imaging regimes in bimodal AFM, and that the discontinuous jumps and contrast reversals are in some way caused by dissipative tip–sample interactions.

The understanding of the different imaging regimes discovered in this work will be of great help to AFM researchers by allowing them to choose their operating parameters intelligently so as to maximize material contrast.

## Supporting Information

The Supporting Information contains two appendices: (1) a detailed description of the tip–sample interaction model used in the simulations and (2) a brief derivation of the kinetic/potential energy of an eigenmode.

File 1 Appendices
