# Supplementary material for: Multiple regimes of operation in bimodal AFM: understanding the energy of cantilever eigenmodes
Source: Beilstein J Nanotechnol. 2013 Jun 21;4:385–93. doi: 10.3762/bjnano.4.45 (PMC3701429; doi:10.3762/bjnano.4.45)
Supplement: File 1 — Appendices [file Beilstein_J_Nanotechnol-04-385-s001.pdf]

Supporting Information

for

Understanding the energy of cantilever eigenmodes: Multiple regimes of operation in bimodal AFM

Daniel Kiracofe*[1,2], Arvind Raman[1] and Dalia Yablon[2]

Address: [1]Purdue University School of Mechanical Engineering, West Lafayette, IN, USA and [2]ExxonMobil Research and Engineering Co., Annandale, NJ, USA

Email: Daniel Kiracofe - daniel.kiracofe@gmail.com, Arvind Raman - raman@purdue.edu, Dalia Yablon - dalia.g.yablon@exxonmobil.com

* Corresponding author

Appendices

Appendix 1: Tip–sample interaction model

In [1] a tip–sample interaction model is suggested, which includes surface energy hysteresis. The model was based on a DMT model. In the original formulation, the attractive and repulsive parts are handled separately (the attractive part was called "long-range dissipative interfacial interactions"). Using a slightly different notation, they can be combined into one expression as follows

$$F_{\mathrm{ts,app}}(d) = \begin{cases} -\frac{HR}{d^2} & d > a_0 \\ \frac{4}{3}E^*\sqrt{R}(a_0-d)^{3/2} - \frac{HR}{a_0^2}, & d < a_0 \end{cases}$$

$$F_{\mathrm{ts,ret}}(d) = \begin{cases} -\frac{(H+4\pi\gamma a_0^2)R}{d^2} & d > a_0 \\ \frac{4}{3}E^*\sqrt{R}(a_0-d)^{3/2} - \frac{HR}{a_0^2} - 4\pi R\gamma, & d < a_0 \end{cases}$$

$$F_{\mathrm{ts}}(d,\dot{d}) = \begin{cases} F_{\mathrm{ts,app}}(d), & \dot{d} < 0 \\ F_{\mathrm{ts,ret}}(d), & \dot{d} > 0 \end{cases}$$

where H, R, a_0, γ are the Hamaker constant, tip radius, intermolecular distance, and surface energy, respectively, E^* is the reduced elasticity $E^* = \left[(1-\nu_{\mathrm{tip}}^2)/E_{\mathrm{tip}} + (1-\nu_{\mathrm{sample}}^2)/E_{\mathrm{sample}}\right]^{-1}$ and ν and E are the Poisson's ratio and Young's modulus of the tip and the sample. We note that this model includes only a single parameter γ, which is the change in surface energy (J/m^2) between approach and retract, to describe the strength of the hysteresis. Two parameters are given in [1], one for $d < a_0$ and a separate one for $d > a_0$. Although that is a more general case, it means that the force described by that model is not necessarily continuous at $d = a_0$. We have restricted ourselves only to the case where the force is continuous.

This model has been shown to match several features of experimental energy dissipation mea-

surements, and it is well suited to analysis (e.g., the method of averaging). However, for numerical simulation it can present a problem. The switch between the approach and retract forces happens instantaneously. This means that the force is discontinuous at the switch, which is clearly nonphysical. This may cause difficulties for the differential equation solver, and it can also introduce nonphysical high-frequency oscillation of the cantilever into the simulation.

Therefore, we suggest a modification that allows the force to be continuous everywhere. Initially, $\dot{d} < 0$ and $F_{\text{ts}}(d) = F_{\text{ts,app}}(d)$. If, at time $t = t_0$ and $d = d_0$, the velocity switches sign from $\dot{d} < 0$ to $\dot{d} > 0$, then the force for time $t > t_0$ is defined as $F_{\text{ts}}(d) = F_{\text{ts,ret}}(d) + \left(F_{\text{ts,app}}(d_0) - F_{\text{ts,ret}}(d_0)\right) e^{-(d-d_0)/\lambda}$, where λ is a decay length (we use 0.1 nm typically). In other words, when the velocity switches, the current trajectory is smoothly transitioned into the new trajectory. The difference between the current and new trajectories decays exponentially. This is illustrated in Figure S1.

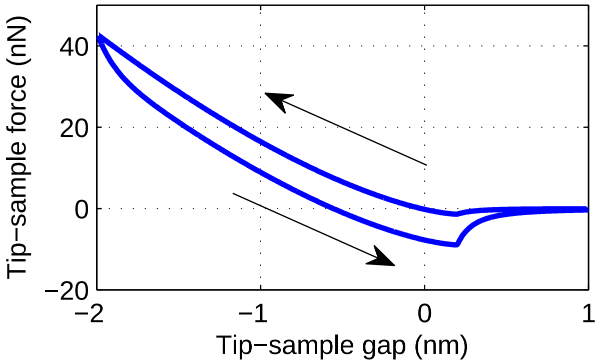

Figure S1: Example of the tip–sample interaction force model used in the simulations.

The above definition is sufficient for single-frequency AFM (e.g., AM-AFM). However, for bimodal AFM, it is possible that the velocity might reverse two (or more) times, and the second reversal might happen before the transition to the new trajectory is complete. Therefore, we instead use this definition: If the velocity reverses at time $t = t_0$ and $d = d_0$, then let F^* be the force at time t_0. Then the force for time $t > t_0$ is defined as $F_{\text{ts}} = F_{\text{ts,m}}(d) + (F^* - F_{\text{ts,m}}(d_0))\, e^{(-d-d_0)/\lambda}$, and "m" corresponds to either "app" or "ret", depending on which direction the velocity has switched to. This allows an arbitrary number of reversals at arbitrary distances, while still always maintaining continuity of the forces.

Appendix 2: Derivation for cantilever energy

By definition, energy is force times displacement. The inertia force on the cantilever is given by $m_i\ddot{q}_i$ and the displacement is q_i. Therefore the kinetic energy at time t is $E_i(t) = \oint m_i\ddot{q}_i dq_i = \int_0^t m_i\ddot{q}_i\dot{q}_i dt$. Assuming a harmonic response at the natural frequency, $q_i = A_i\cos(\omega_i t + \phi)$, then $\dot{q}_i = -\omega_i A_i \sin(\omega_i t + \phi)$ and $\ddot{q}_i = -\omega_i^2 A_i \cos(\omega_i t + \phi)$. So $E_i(t) = m_i\omega_i^3 A_i^2 \int_0^t \sin(\omega_i t + \phi)\cos(\omega_i t + \phi)dt$=$m_i\omega_i^2 A_i^2\cos^2(\omega_i t)/2$. The maximum kinetic energy over the cycle is $m_i\omega_i^2 A_i^2/2 = k_i A_i^2/2$.

The spring force on the cantilever is given by $k_i q_i$. Therefore the potential energy at time t is $E_i(t) = \oint k_i q_i dq_i = \int_0^t k_i q_i \dot{q}_i dt$. Using a similar derivation, it can be shown that the maximum kinetic energy over the cycle is equal to the maximum potential energy over the cycle (the maximums happen at different times). Therefore, the body of the paper could have been formulated in terms of the potential energy stored in each eigenmode with no change in the conclusions.

References

1. García, R.; Gómez, C. J.; Martinez, N. F.; Patil, S.; Dietz, C.; Magerle, R. *Phys. Rev. Lett.* **2006**, *97*, 016103. doi:10.1103/PhysRevLett.97.016103.
